# Superexchange Mechanism in Coupled Triangulenes Forming Spin-1 Chains

**DOI:** 10.1021/acs.nanolett.4c01604

**Published:** 2024-06-05

**Authors:** Yasser Saleem, Torben Steenbock, Emha Riyadhul Jinan Alhadi, Weronika Pasek, Gabriel Bester, Pawel Potasz

**Affiliations:** † Institut für Physikalische Chemie, Universität Hamburg, Grindelallee 117, D-20146 Hamburg, Germany; ‡ Institute of Physics, Faculty of Physics, Astronomy and Informatics, Nicolaus Copernicus University, Grudziadzka 5, 87-100 Toruń, Poland

**Keywords:** spin, magnetism, graphene, quantum dots, two-dimensional materials

## Abstract

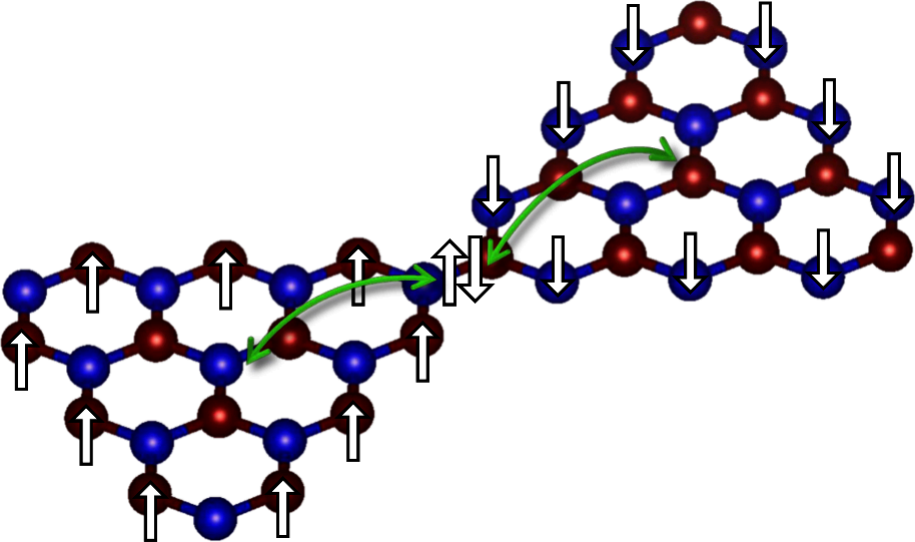

We show that the origin of the antiferromagnetic coupling in spin-1 triangulene chains, which were recently synthesized and measured by Mishra et al. (Nature2021, 598, 287−29234645998 10.1038/s41586-021-03842-3), originates from a superexchange mechanism. This process, mediated by intertriangulene states, opens the possibility to control parameters in the effective bilinear-biquadratic spin model. We start from the derivation of an effective tight-binding model for triangulene chains using a combination of tight-binding and Hartree–Fock methods fitted to hybrid density functional theory results. Next, correlation effects are investigated within the configuration interaction method. Our low-energy many-body spectrum for *N*_Tr_ = 2 and *N*_Tr_ = 4 triangulene chains agree well with the bilinear-biquadratic spin-1 chain antiferromagnetic model when indirect coupling processes and superexchange coupling between triangulene spins are taken into account.

The antiferromagnetic (AFM) Heisenberg spin-1 chain exhibits intriguing properties, including symmetry-protected topological order and a gapped excitation spectrum known as the Haldane gap.^1−4^ Furthermore, in finite systems, characteristic spin  edge excitations emerge.^5^ Affleck, Kennedy, Lieb, and Tasaki (1987) proposed the AKLT model to elucidate the origin of the Haldane gap, introducing an isotropic one-dimensional spin-1 model with a unique ground state that also confirms the presence of an energy gap to excited states.^3^ This model, characterized by an additional biquadratic exchange term, allows for a representation of the ground state on a valence-bond basis. The AFM Heisenberg spin-1 chain, along with models like AKLT, belongs to a broader class of bilinear-biquadratic (BLBQ) spin-1 chain models, which display diverse magnetic orderings dependent on the energetic ratio between linear and biquadratic exchange terms (see refs (6−8) and references therein).

Spin models are effective models of the low-energy subspaces of a Fermionic system.^9−11^ They can be derived from other simplified models such as the Hubbard model, which is an approximation of the full interacting Hamiltonian containing all two-body Coulomb interactions. AFM coupling in spin models can be explained by the kinetic exchange mechanisms, a coupling between singly and doubly occupied sites by effective hopping. This process favors AFM ordering of electron spins, since electrons with opposite spins are able to freely hop from one site to the next without being repelled due to Pauli exclusion.^12^ Another exchange mechanism, known as superexchange has been proposed to explain AFM order in transition metal oxides.^12−15^ In that case, two cation orbitals are separated by an anion. As such, direct hopping between cations is quenched, while hopping between localized d-orbitals (located on the cation) occurs through an intermediate p-state (located on the anion).^12,14,16−19^

Recently, in 2021 Mishra et al.^20^ observed signatures of fractional edge states and gapped spin excitations in spin-1 triangulene chains. An AFM coupling between neighboring spins was described as a direct process in an effective Hubbard model with an enhanced next-next-nearest-neighbor (NNNN) hopping commonly labeled *t*_3_ in literature.^10,20,21^ While that model captures major physical properties of the system, we show that the microscopic mechanism of the AFM coupling is an indirect superexchange process that includes states localized on the connection of triangulene molecules (intertriangulene states). This opens up opportunities to control the parameters of the spin model.

In our work, we start from a combination of tight-binding (TB) and Hartree–Fock (HF) methods and fit the results to density functional theory (DFT), in order to derive an effective single-particle model of triangulene chains that includes the presence of valence band electrons. The obtained spatial variation of TB hopping integrals takes into account geometric effects. We include correlations within the complete active space (CAS) by using the configuration interaction (CI) method. We analyze the low-energy many-body spectrum as a function of CAS size and compare it with experimental results from ref (20). We find that the direct kinetic exchange contribution is too small to explain the spin-1 AFM model spectrum, and the exchange mechanism that dominates the value of the coupling constant *J* is due to superexchange. Finally, we fit the many-body spectrum to the BLBQ model for *N*_Tr_ = 2 and *N*_Tr_ = 4 triangulene chains, confirming that, at low energies, the system behaves as a spin-1 chain and allows us to extract effective spin parameters.

Triangular graphene quantum dots (TGQDs) with zigzag edges exhibit promising potential as building blocks for spin chains. This is because the single-electron spectrum for these quantum dots collapses to a degenerate shell at the Fermi level, a phenomenon resulting from sublattice imbalance. Consequently, it is predicted that these quantum dots possess a magnetic spin-polarized ground state (GS) at half-filling,^22−30^ a property consistent with Lieb’s theorem.^31^ The degeneracy of this shell is proportional to the length of a single edge of the triangular structure.^27^ Triangulene, which is the second smallest TGQD with zigzag edges, containing 22 carbon atoms (also referred to as Clar’s hydrocarbon), is of particular interest in this work and is shown in Figure 1(a). Triangulene has two degenerate states at the Fermi level and consequently possesses a spin-1 ground state. It was predicted to exist and attempted to be synthesized in 1953 by Clar and Stewart.^32^ Triangulene faced synthesis challenges due to the molecule’s high reactivity arising from the two unpaired electrons. Overcoming this challenge more than 60 years later, Pavliček et al.^33^ successfully synthesized triangulene in 2017. Following this milestone, noteworthy experimental work focused on the synthesis of triangulene as well as larger-sized TGQDs emerged.^34−36^

**Figure 1:**
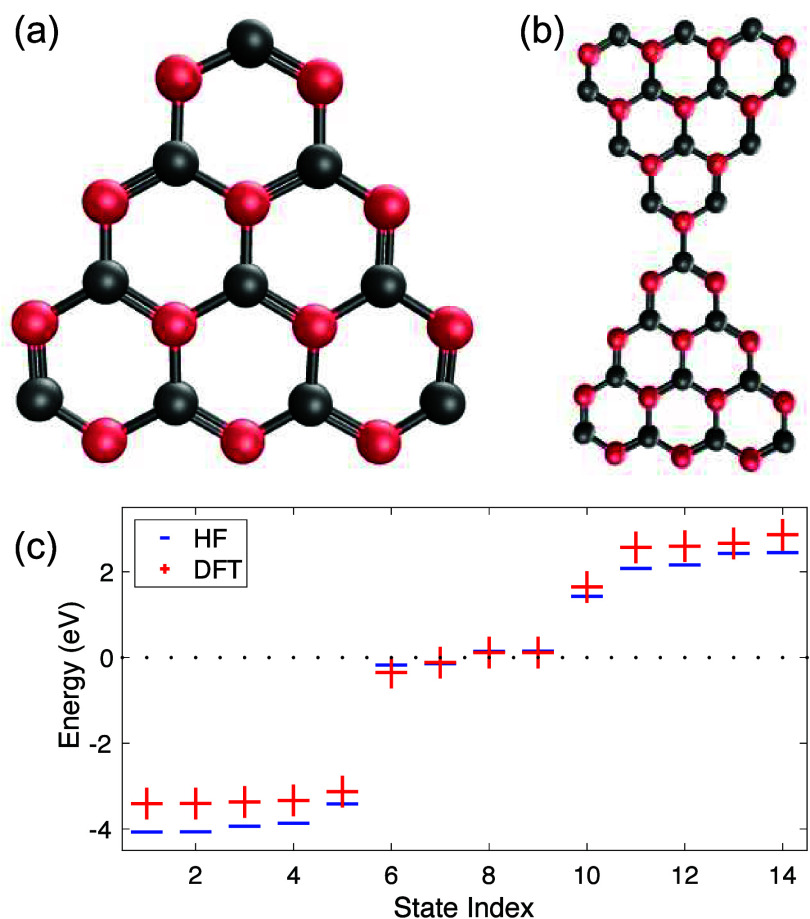
Geometrical representation of (a) the triangulene and (b) the triangulene dimer. The balls denote location of carbon atoms with the red balls corresponding to sublattice A and the gray balls corresponding to sublattice B. The sticks connecting carbon atoms refer to the alternating single and double bonds. In (c) we show the single-particle spectrum of the triangulene dimer for states near the Fermi level. The blue horizontal lines are HF results, while the red crosses correspond to DFT results obtained with the B3-LYP exchange–correlation functional. The dotted line corresponds to the Fermi level at half-filling.

The first step in constructing a synthetic spin-1 chain using triangulene involved combining two triangulene molecules to form a triangulene dimer (TD), as illustrated in Figure 1(b). This was accomplished experimentally^20,37^ and investigated theoretically^9,10,38,39^ in recent years. To understand the single-particle properties of the TD, we employ a p_*z*_ orbital TB model, akin to previous studies.^10,40−42^ One assumption in these TB models is that the hopping parameters between two sites depend solely on the distance between them. However, when graphene is cut into a finite flake, electron hopping can be dramatically modified, especially along the edge. We correct the hoppings by solving the HF equation given by^28,29,41^
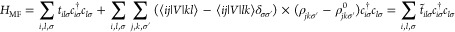
1where *c*_*i*,σ_^†^/*c*_*i*,σ_ creates/annihilates a p_*z*_ electron on site *i* with spin σ. *t*_*ilσ*_ is the two-dimensional (2D) infinite system hopping elements. We take the following standard accepted values for *t*_*ilσ*_: *t* = −2.8 eV as the nearest-neighbor (NN) hopping, *t′* = −0.1 eV as the next-nearest-neighbor (NNN) hopping, and *t*_3_ = −0.07 eV is the NNNN hopping.^43^ ρ_*jkσ′*_^0^ are the density matrix elements for a 2D infinite graphene sheet obtained previously.^28,41^ ρ_*jkσ′*_ is the density matrix element for the finite system computed with respect to the HF ground state. The Coulomb matrix elements ⟨*ij*|*V*|*kl*⟩ are screened by a dielectric constant κ = 3, which gives good agreement with DFT calculations, as seen in Figure 1; see details of calculations in the Supporting Information.^44^

For the TD shown in Figure 1(b), there are *N*_s_ = 44 atomic sites, and in the single-orbital model here, this corresponds to *N*_e_ = 44 electrons for the charge neutral system. It is convenient, for the CI calculations that will follow, to remove four electrons from the TD, similar to what was done in previous work,^26,28,29^ and add them back when performing CI calculations. Therefore, we self-consistently solve the HF equation, as represented in eq 1, for *N*_*↑*_ = 20 spin-up and *N*_*↓*_ = 20 spin-down p_*z*_ electrons, which is justified by noticing the presence of the energy gap between the lowest *N*_st_ = 20 energy states and four degenerate shell states, seen in the tight-binding spectrum.^10^ We obtained HF quasiparticle orbitals and compared them with the results from DFT calculations for a + 4 cation TD. Figure 1(c) shows agreement between DFT and HF energy levels, and Figure 2 shows agreement between their corresponding wave functions. We use one fitting parameter between the two calculations, which is a static screening constant κ = 3, which reduces the strength of the Coulomb interaction by 1/κ. Examination of Figure 1(c) reveals the presence of four distinct states in the proximity of the Fermi level, and a substantial energy gap separates these states from others. The wave functions of states No. 5 (the first state below the degenerate shell, Figure 2(a),(g)) and No. 10 (the first state above the degenerate shell, Figure 2(f),(l)) are localized at the connection between the two triangulenes; we call them intertriangulene states. The wave functions of four degenerate shell states significantly differs from the wave functions of two degenerate states from an isolated triangulene, as illustrated in Figure 2(b)–(e) for the HF calculations and Figure 2(h)–(k) for DFT calculations (compared with the Figure S1 inset in the Supporting Information(44)). In particular, after HF self-consistent calculations, the degenerate shell states No. 6 (Figure 2(b),(h)) and No. 7 (Figure 2(c),(i)) strongly hybridize with the intertriangulene states (compared with densities from the TB wave functions shown in Figure S8 of the Supporting Information). This implies that, in order to analyze coupling between the spins of isolated trianglulenes, we must take into account at least the six states shown in Figure 2.

**Figure 2:**
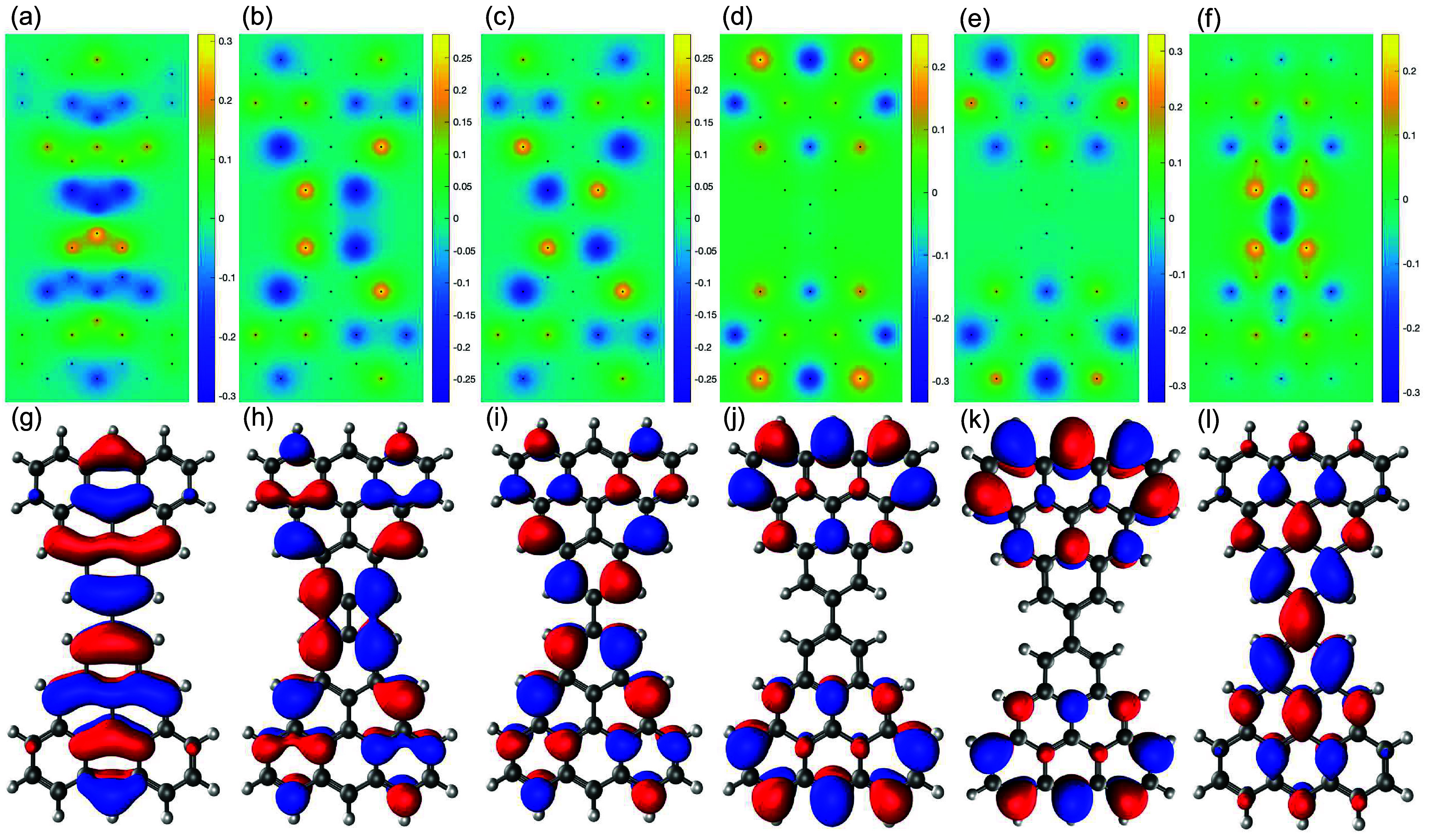
Hartree–Fock (a)–(f) and DFT (g)–(l) wave functions for the six states located closest the Fermi level at half-filling. (a)–(f) and (g)–(l) corresponds to states 5–10 shown in Figure 1(c).

As seen, in the last line of eq 1, the HF equation reduces to a single effective hopping parameter, *t̃*_*ilσ*_ which accounts for finite-sized modifications of the TB hoppings. It is given explicitly as
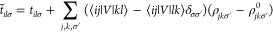
2It is apparent in eq 2 that, when ρ_*jkσ′*_ = ρ_*jkσ′*_^0^, the effective hopping parameter reduces to that of the extended system hopping parameters *t*_*ilσ*_. Furthermore, deviations of the hopping elements will largely depend on the density matrix elements. As such, to obtain effective hopping parameters that more closely resemble those that should be used in a TB model, we recompute the density matrix ρ_*jkσ′*_ at charge neutrality (a detailed analysis of obtaining effective hopping elements is discussed in the Supporting Information). Thus, all states below the dotted line in Figure 1(c) are occupied. Figure 3 shows how the extended system hopping parameters are modified in HF for the TD. The line connecting two atoms represents the hopping between the two sites, while the color represents the magnitude of the hopping. We see in Figure 3(a) that, in the center of the structure, the effective NN hopping is almost equal to the 2D infinite system value *t*. Away from the center is where the largest modifications occur. There is an enhancement of the NN hopping element of about 10% near the bottom of each triangulene molecule and a reduction of about 25% on the connection between the two triangulenes. Similarly, the NNN *t′* hopping element in the center of each individual triangulene resembles that of the 2D infinite system, but a 300% enhancement of the NNN hopping element along the bottoms of each triangulene molecule is observed, as seen in Figure 3(b). Finally in Figure 3(c), even though we observe an overall enhancement of the NNNN *t*_3_ hopping, we observe a reduction of it on the connection between the two triangulene molecules. This contradicts a strong enhancement of *t*_3_ in the effective Hubbard model considered in previous works.^10,20,45^ We notice that the renormalization of the hopping elements is dominantly attributed to the lack of charge uniformity brought upon by the edge effects of the TD.

**Figure 3:**
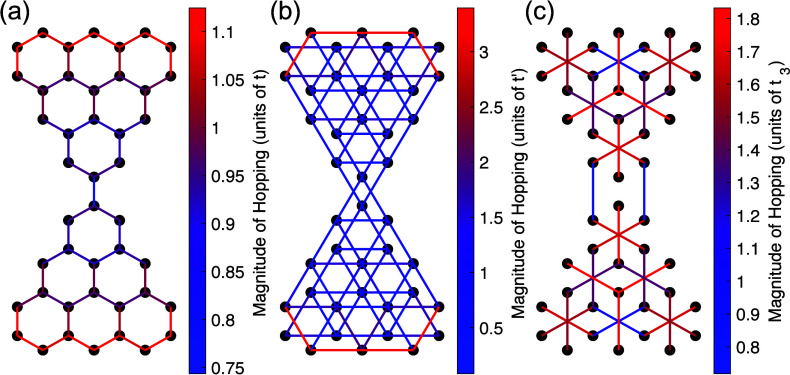
Charge neutral effective HF hopping elements for (a) nearest neighbor *t*, (b) next nearest neighbor *t′*, (c) next–next nearest neighbor *t*_3_. The black dots correspond to the locations of carbon atoms, and the line connecting them corresponds to the effective hopping between carbon atoms. The units for each color plot are measured with respect to their 2D infinite system hopping values.

In order to include the effects of correlations and obtain excited states of the TD we combine CI with the HF method. By rotating the many-body Hamiltonian to the basis of HF states one obtains^29,46^
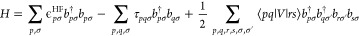
3where ϵ_*pσ*_^HF^ are the energies of the HF quasiparticle orbitals obtained by solving eq 1. *b*_*p*,σ_^†^/*b*_*p*,σ_ creates/annihilates an electron on the HF orbital p with spin σ. The double counting corrections τ_*pqσ*_ = ∑_*m*,*σ′*_[⟨*pm*|*V* |*mq*⟩ – ⟨*pm*|*V* |*qm*⟩]*n*_*mσ′*_, with *n*_*mσ′*_ being HF occupations, arise to avoid double counting of the interactions that were already contained at the mean-field level.^29,46^ This effectively lowers the contribution of the last term in eq 3.

To solve the problem exactly for *N*_Tr_ = 2, one would need to include *N*_e_ = 44 electrons on *N*_st_ = 44 states, which is currently impossible to solve due to an unmanageably large many-body Hilbert space. Thus, we solve the CI Hamiltonian given by eq 3 on the basis of configurations by considering CAS(4,4), CAS(6,6), CAS(10,10), and CAS(14,14) states, as shown in Figure 4(a), where we use the notation CAS(*N*_st_,*N*_e_) with *N*_st_/2 taken above and *N*_st_/2 taken below the dotted line shown in Figure 1(c).

**Figure 4:**
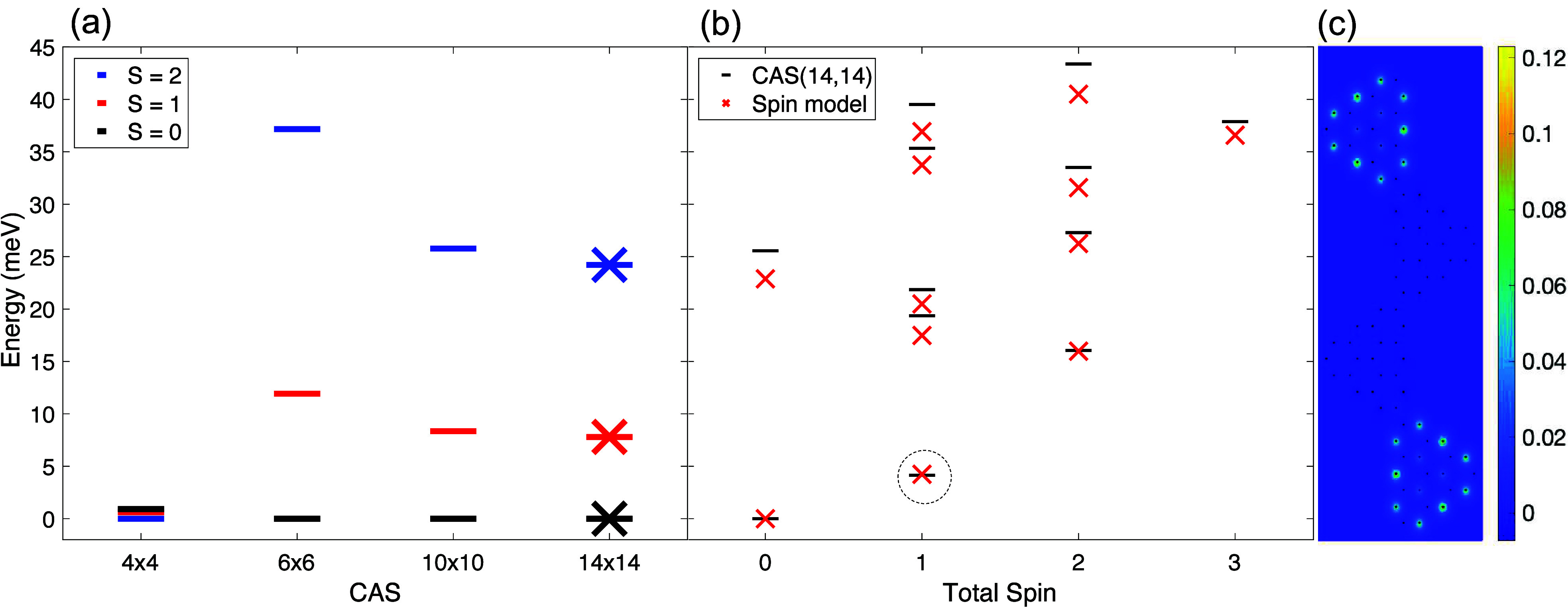
(a) Many-body spectrum for *N*_Tr_ = 2 triangulenes as a function of CAS size. The *x*-axis denotes the number of states taken relative to the dotted line shown in Figure 1(c); i.e., six states means taking 3 states above and 3 states below the dotted line. The “x”” data points overlaying the data for 14 states correspond to the spin model results for *J* = 8.21 meV and β = 0.016. (b) Comparison of the many-body spectrum for *N*_Tr_ = 4 triangulenes with corresponding BLBQ spin model results. In (c), we show the many-body spin density for the lowest energy triplet state for *N*_Tr_ = 4 indicated by a circle in (b).

In the CAS(4,4) results, the ground state is ferromagnetic (FM) with *S* = 2 and corresponds to a Hund’s rule like filling of electrons on the degenerate shell consisting of four states. This occurs when the Coulomb exchange among electrons on the shell of four states dominates compared to the effective AFM exchange coupling *J* between the spin-1 quasiparticles. While this contradicts Lieb’s theorem for the Hubbard model, we consider a many-body Hamiltonian with terms that violate bipartite conditions of the lattice, so the ground state need not be a singlet. When we include, in our active Hilbert space, the intertriangulene HF states, *N*_st_ = 6, shown in Figure 2(a),(f), we observe the ground state transitions from FM to AFM. This AFM ground state agrees with recent experiments.^20^ Electrons from the degenerate shell states can now effectively hop from one triangulene molecule to the neighboring one via the intertriangulene states (states 5 and 10 from Figure 1(c)). Thus, AFM superexchange is needed in order to explain the spin-1 chain character recently observed experimentally.^20^ Further increase of the CAS Hilbert space size only quantitatively corrects the values of the energy gaps between the singlet *S* = 0, triplet *S* = 1, and quintuplet *S* = 2. The singlet–triplet gap measured experimentally^20^ is comparable to the results for CAS(14,14) (14 and 8 meV, respectively) and could be improved by using a more advanced model for the dielectric screening.

We fit our CAS(14,14) spectrum to the BLBQ model given by
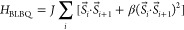
4where *J* is the bilinear exchange coupling constant between neighboring spin sites and β is its biquadratic partner. By fitting to the BLBQ model, we extract the effective exchange terms *J* = 8.21 meV and β = 0.016. Finally, in Figure 4(b) we compare many-body spectra for *N*_Tr_ = 4 [we take all eight degenerate shell states (see Figure S3 states labeled B) and six intertriangulene states (see Figure S3 labeled A and C) in the calculations] and the corresponding energy spectrum of the spin Hamiltonian given by eq 4. We find good agreement between energy spectra in both models for each total spin eigenstate, proving the validity of the spin-1 model description (with almost the same parameters as for the TD case, the effective exchange terms are *J* = 8.29 meV and β = 0.016). Discrepancies between our computed spin parameters and the experimentally measured spin parameters (*J* = 18 meV and β = 0.09)^20^ could be attributed to levels of approximation in the theory. One such is our use of a static dielectric screening model. Experimentally, the samples of triangulene chains lie on a gold substrate, and the effects of screening due to this substrate are not well understood. The gold substrate might be a crucial issue here because it quenches the long-range part of the Coulomb interaction, and the realistic model would be closer to a short-range Hubbard model. As we show in the Supporting Information, Figure S2, for the Hubbard model, both *J* and β are substantially increased compared to the full model calculations.

Small differences in the excited-state energies can be attributed to a finite Hilbert space. We note that calculations that include more HF states are beyond our numerical capabilities. Figure 4(c) shows the many-body spin density for the lowest energy triplet state, which resembles characteristic edge states in the Haldane phase. It is worth mentioning that the excess of this spin density is similar to the one from the isolated triangulene (see the Supporting Information(44)).

The AFM superexchange mechanism is usually derived using higher order perturbation theory. We notice that, after self-consistent HF calculations, we already are in a basis of strongly hybridized states of the degenerate shell and the intertriangulene states. This hybridization comes from interactions between electrons populating all filled states and virtual electrons on a degenerate shell and thus cannot be uncoupled by any rotation within the effective low-dimensional Hilbert space; one needs to include all the single-particle energy states, not only the four degenerate shell states (for *N*_Tr_ = 2) and intertriangulene valence and conduction band states. This makes it difficult to explicitly identify the superexchange processes responsible for the antiferromagnetic coupling between two *S* = 1 spins within perturbation theory. The degenerate states from isolated triangulene molecules are significantly different than the four HF degenerate shell states in the TD. We extend the discussion about identification of superexchange mechanism in the Supporting Information.^44^

The presence of indirect coupling between spins in triangulene spin-1 chains opens a new possibility to control parameters in the BLBQ model given by eq 4. While it might be difficult, or even impossible, to go beyond the Haldane phase on the phase diagram,^8^ slight variations of β might be possible by tuning the energy gap between the intertriangulene states and the degenerate shell states, which translates into control of the singlet–triplet splitting. The superexchange mechanism discussed in our work for triangulene spin-1 chains should also be responsible for AFM coupling in other triangulene systems,^47,48^ in particular, in recently proposed two-dimensional triangulene crystals.^39,49^ Following this path, such newly created AFM spin–lattice systems should reveal similarities to antiferromagnetism in transition metal oxides,^16,17,19^ where the superexchange mechanism plays a dominant role. Superexchange interaction in anion-bridged dinuclear transition metal complexes can be described by the Goodenough–Kanamori rules.^50−52^ In analogy to these systems, it might be possible to establish similar rules for understanding the spin coupling in triangulene spin chains. However, the originating from the degenerate shell of triangulene are significantly different than the d-orbitals of the metal ions. Thus, determining whether similar rules apply here requires more detailed studies.

## Supporting Information Available

Details of the DFT and Coulomb matrix elements calculations, extension of CI calculations within different many-body Hamiltonians, details of spin model calculations, analysis of the superexchange mechanism, derivation of effective HF hopping elements (PDF)
