# Supplementary material for: Superexchange Mechanism in Coupled Triangulenes Forming Spin-1 Chains
Source: Nano Lett. 2024 Jun 5;24(24):7417–23. doi: 10.1021/acs.nanolett.4c01604 (PMC11194845; doi:10.1021/acs.nanolett.4c01604)
Supplement: Supplementary file 1 — nl4c01604_si_001.pdf [file nl4c01604_si_001.pdf]

Supporting Information
Superexchange Mechanism in Coupled Triangulenes Forming spin-1 Chains

Yasser Saleem,[1] Torben Steenbock,[1] Emha Riyadhul Jinan Alhadi,[2] Weronika Pasek,[2] Gabriel Bester,[1] and Pawel Potasz[2]

[1]*Institut für Physikalische Chemie, Universität Hamburg, Grindelallee 117, D-20146 Hamburg, Germany*
[2]*Institute of Physics, Faculty of Physics, Astronomy and Informatics, Nicolaus Copernicus University, Grudziadzka 5, 87-100 Toruń, Poland*

(Dated: May 27, 2024)

I. DENSITY FUNCTIONAL THEORY

All density functional theory (DFT) calculations were carried out with the TURBOMOLE 7.5 program package[1] employing the B3LYP global hybrid exchange–correlation functional with 20% of the exact Hartree-Fock exchange[2–7], Ahlrich's triple-zeta split-valence basis set with polarization functions on all atoms, def2-TZVP[8], and the empirical dispersion correction of Grimme in the third generation[9]. Additionally, we employ the multipole-accelerated resolution of identity approximation for Coulomb integrals (MARIJ)[8,10–12] to speed-up the calculation of the Coulomb integrals in the self-consistent field (SCF) algorithm.

First, we perform structure optimizations on the spin ground states approximated by Broken-Symmetry (BS) determinants[13], where the $S = 1$ spins of adjacent triangulenes are aligned anti-parallel. In the structure optimizations convergence criteria of 10^{-7} Hartree for the energy and 10^{-4} Hartree/Bohr for the gradient were employed. On top of the optimized structures, we perform single-point calculations for non-spin polarized cations that serve as the reference in the full configuration interaction (CI) calculations. The cations are obtained by removing all unpaired electrons, two for each triangulene subunit, from the systems. In these single-point calculations,the same convergence criterion for the energy of 10^{-7} Hartree is employed.

II. CALCULATION OF COULOMB MATRIX ELEMENTS IN THE SITE BASIS

The Coulomb matrix elements $\langle ij|V|kl\rangle$ are given explicitly as[14]

$$\langle ij|V|kl\rangle = \int\int d\vec{r}_1 d\vec{r}_2 \phi_i^*(\vec{r}_1)\phi_j^*(\vec{r}_2)\frac{2R_y}{\kappa|\vec{r}_1-\vec{r}_2|}\phi_k(\vec{r}_2)\phi_l(\vec{r}_1), \tag{1}$$

where, $\vec{r}_1$, $\vec{r}_2$ are the coordinates of electron 1 and electron 2, κ is the dielectric constant, and R_y is the Rydberg constant. $\phi_i(\vec{r})$ are p_z Slater orbitals centered on atom i, they are given as

$$\phi_i(\vec{r}) = \left(\frac{\xi^5}{32\pi}\right)^{\frac{1}{2}} z e^{-\frac{\xi}{2}|\vec{r}-\vec{r}_i|}, \tag{2}$$

where $\vec{r}_i$ is the position of carbon atom i, and $\xi = 3.25$[15]. The lengths are in units of Bohrs. The integrals are solved efficiently in real space using the VEGAS integration alogorithim contained in the GNU scientific library[16]. Table I shows all quantum mechanical scattering elements considered and their magnitude for $\kappa = 1$. When electrons are far enough away, we take a classical limit of the Coulomb elements, mainly we also take the elements: $\langle ij|V|ji\rangle = \frac{2R_y}{|\vec{R}_i-\vec{R}_j|}$ for atoms i,j beyond NNN.

Element	Value (eV)
$\langle 1,1\|V\|1,1\rangle$	17.311
$\langle 1,2\|V\|2,1\rangle$	8.942
$\langle 1,3\|V\|3,1\rangle$	5.583
$\langle 1,1\|V\|1,2\rangle$	3.027
$\langle 1,2\|V\|3,1\rangle$	1.664
$\langle 1,2\|V\|1,2\rangle$	0.774
$\langle 1,1\|V\|2,2\rangle$	0.774
$\langle 2,2\|V\|1,3\rangle$	0.535
$\langle 1,2\|V\|2,3\rangle$	0.356
$\langle 1,1\|V\|1,3\rangle$	0.306
$\langle 1,1\|V\|1,4\rangle$	0.113
$\langle 1,2\|V\|2,4\rangle$	0.113
$\langle 1,3\|V\|3,4\rangle$	0.122

TABLE I. Table of scattering Coulomb matrix elements of p_z electrons for $\kappa = 1$. The numbers 1,2,3, and 4 correspond to atoms where the difference in the numbers corresponds to the neighbor distance i.e. a difference of 1 corresponds to NN, a difference of 2 between numbers corresponds to NNN, and a difference of 3 corresponds to NNNN.

III. CONFIGURATION INTERACTION BASIS

The many-body Hilbert space can be divided into smaller subspaces with total spin **S** and azimuthal spin S^z. We construct the basis, in the occupation number representation, distributing particles among single-particle states labeled with spin S^z. The total number of possible configurations N_{cf} for N_{e} particles distributed on N_{st} single particle states with a given spin $N_\downarrow$ or $N_\uparrow$,

where $N_e = N_\downarrow + N_\uparrow$ is determined by a product of binomial coefficients, $N_{cf} = \binom{N_{st}}{N_\uparrow} \cdot \binom{N_{st}}{N_\uparrow}$. We do not rotate the Hamiltonian matrix to a $\mathbf{S}$ basis as this is an additional computational cost, and instead determine the ground state from calculations of expectation value of total spin S for each energy eigenstate.

IV. TRIANGULENE

We analyze a single triangulene molecule, a triangular graphene quantum dot with zigzag edges shown in Fig. 1(a) of the main text. This quantum dot has broken sublattice symmetry as seen by counting the number of red balls (carbon atoms belonging to sublattice A) and the number of gray balls (carbon atoms belonging to sublattice B). This is also a bipartite lattice, and as such, Lieb's theorem applies[17].

We start by performing DFT calculations, and find the ground state to be $S = 1$ in agreement with Lieb's theorem and previous experimental and theoretical work[18–20]. We then perform HF calculations by solving the HF equation. We take $\kappa = 3$ giving the results shown in Fig. S1. At the top of the VB there are two degenerate states that are spin up, and a large gap that separates the spin down states. The splitting between the spin up and spin down states arises from a net spin-polarization. These states are localized at the edge, and the spin density of triangulene (shown as an inset in Fig. S1) shows the localized spin-1 quasi particle tends to localize at the edge of the triangle.

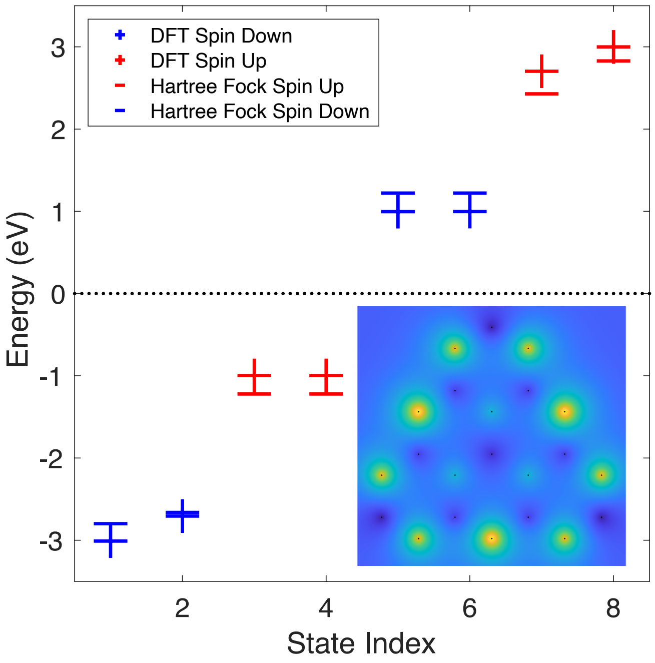

FIG. S1. Single-particle spectrum of triangulene $N_{Tr} = 1$ at half-filling. The horizontal bars are HF results, while the crosses correspond to DFT results. Red color corresponds to spin up levels while blue color corresponds to spin down levels. The inset shows the spin density of the HF ground state.

V. COMPARISON BETWEEN DIFFERENT MANY-BODY INTERACTING FERMIONIC MODELS - RESULTS FOR TWO TRIANGLES

We compare results obtained within different many-body Hamiltonians by restricting Coulomb matrix elements in real space to the dominant ones. Within the Hubbard model, one gets an effective mean-field Hamiltonian given as

$$H_{MF}^{H} = \sum_{i,l,\sigma} t_{il\sigma} c_{i\sigma}^\dagger c_{l\sigma} + U \sum_{i,\sigma} \left(\langle \hat{n}_{i,-\sigma} \rangle - \frac{1}{2} \right) \hat{n}_{i\sigma}, \quad (3)$$

where the Hubbard parameter $U = \langle ii|V|ii \rangle$, $\hat{n}_i = c_{i\sigma}^\dagger c_{i\sigma}$, and $\langle \hat{n}_{i,-\sigma} \rangle = \rho_{ii,\sigma'}$, are diagonal elements of density matrix with $\sigma' = -\sigma$. For the extended Hubbard model one has

$$H_{MF}^{EH} = \sum_{i,l,\sigma} t_{il\sigma} c_{i\sigma}^\dagger c_{l\sigma} + U \sum_{i,\sigma} \left(\langle \hat{n}_{i,-\sigma} \rangle - \frac{1}{2} \right) \hat{n}_{i\sigma} + \sum_{i \neq j,\sigma,\sigma'} V_{ij} \left(\rho_{jj\sigma'} - \frac{1}{2} \right) \hat{n}_{i\sigma}, \quad (4)$$

where $V_{ij} = \langle ij|V|ji \rangle$. The Coulomb matrix elements in the basis of mean-field energies $\langle pq|V|rs \rangle$ are obtained using a basis rotation

$$\langle pq|V|rs \rangle = \sum_{i,j} V_{ij} B_{ip}^* B_{jq}^* B_{jr} B_{is}, \quad (5)$$

where $b_{p\sigma} = \sum_i B_{ip} c_{i\sigma}$, and B_{ip} are eigenvectors obtained by solving the mean-field Hamiltonian. The form of the many-body Hamiltonian is the same for Hubbard and extended Hubbard, and full interacting models given by Eq. 3 in the main article, but the models differ in the parameters used defined by $\epsilon_{p\sigma}^{HF}$, $\tau_{pq\sigma}$ and Coulomb elements given by Eq. 5. A real space spin density distribution for a given many-body state D, used when computing the spin density shown in Fig. 4 in the main article, is calculated using the formula

$$\langle D|\hat{n}_{i\sigma}|D \rangle = \sum_{p,q} B_{pi}'^* B_{qi}' \langle D|b_{p\sigma}^\dagger b_{q\sigma}|D \rangle = \sum_{p,q} B_{pi}'^* B_{qi}' \sum_{c,d} A_c^*(D) A_d(D) \langle c|b_{p\sigma}^\dagger b_{q\sigma}|d \rangle , \quad (6)$$

where $|D\rangle = \sum_c A_c |c\rangle$ and A_c are expansion coefficients, $|c\rangle = \prod_{p,\sigma} b_{p\sigma}^\dagger |0\rangle$ are occupation configuration states and $|0\rangle$ is our vacuum state corresponding to HF state with all valence band states filled (all states below CAS). B_{pi}' are coefficients from inverse transformation, $c_{i\sigma} = \sum_p B_{pi}' b_{p\sigma}$, where $B_{pi}' = B_{ip}^*$.

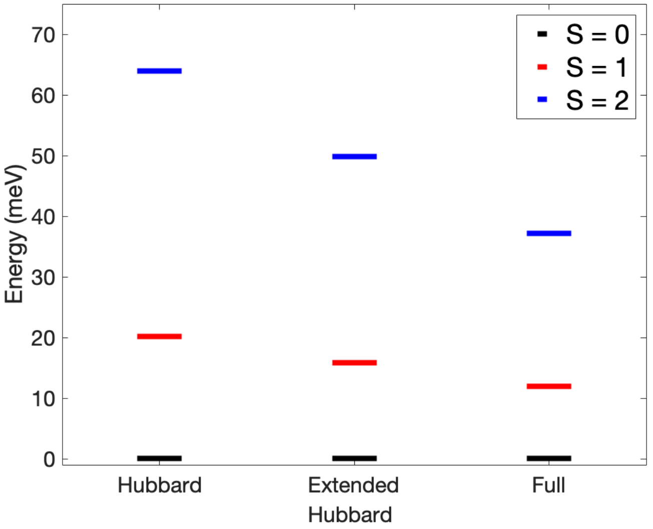

FIG. S2. Many-body spectrum obtained for the Hubbard model, extended Hubbard model, and fully interacting model for CAS(6,6) calculations. Hubbard model gives $\beta = 0.025$ and $J = 21.85$ meV, the extended Hubbard model gives $\beta = 0.023$ and $J = 15.82$ meV, and the full model gives $\beta = 0.018$ and $J = 10.67$ meV.

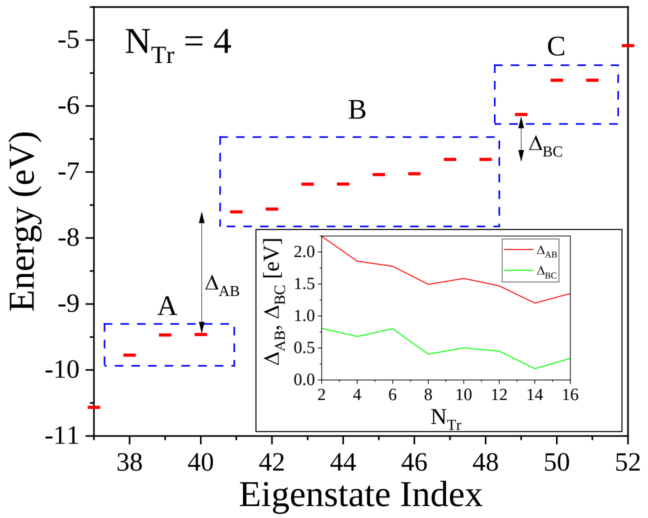

FIG. S3. Hartree-Fock spectrum from the extended Hubbard model for $N_{\mathrm{Tr}} = 4$. Three sets of states, A, B, and C are indicated, with energy gaps between them, Δ_{AB} and Δ_{BC}. The inset shows the scaling of the energy gaps with the number of triangulenes N_{Tr} in a chain.

Fig. S2 shows the many-body spectrum for the Hubbard model, extended Hubbard model and the fully interacting model for $N_{\mathrm{Tr}} = 2$ structure. Calculations in each case were done for CAS(6,6). We find that the Hubbard model tends to overestimate the spin gaps, with the extended Hubbard model capturing most of the quantitative features of the full model. Notice that we are showing only the three lowest energy states, as the excited states are separated from these states by a large gap on the order of hundreds of meV.

VI. EXTENDED HUBBARD MODEL RESULTS FOR $N_{\mathrm{Tr}} = 4$ TRIANGULENES

Using the extended Hubbard model, we analyze the $N_{\mathrm{Tr}} = 4$ structure and discuss properties of longer triangulene chains. In Fig. S3, we show the Hartree-Fock energy spectrum indicating the energy gaps between the inter-triangulene states (sectors B) and the degenerate shell states (sector A and C), Δ_{AB} and Δ_{BC}. Within this model, the degenerate shell states split into a set of four double degenerate states. There are three inter-triangulene states below and three above the degenerate shell. The energy gap Δ_{AB} is around twice smaller than Δ_{BC}, and this relation is approximately true also for longer chains, see the inset. These gaps determine the role of excitations and need to be related to scattering Coulomb matrix elements. For $\kappa = 3$ used here, we find some of scattering elements between sector A and B, and between B and C as large as 0.2 eV (e.g. $\langle AA|v|BB\rangle$ and $\langle AB|v|BC\rangle$). Comparing them to energy gaps $\Delta_{\mathrm{AB}} \sim 1.8$ eV, $\Delta_{\mathrm{BC}} \sim 0.7$ eV, one can conclude that the states from sector C are as important as the states from sector A, see also Section VIII, where we discuss the superexchange mechanism.

Fig. S4 shows charge densities in these three energy sectors; the densities are normalized by the number of states in a given sector. Sector A has localized charge density in the center of the chain, the central inter-triangulene connection and on the first and last triangulenes. On can notice a higher charge density on carbon atoms from the same sublattice as the degenerate shell states, which confirms hybridization with states from sector B (within a tight-binding model, charge density from sector A is similar to that from sector C). In sector B charge density is mainly localized on the edges of triangulenes, and only one sublattice. Sector C has charge density on the three connections between the four triangulenes.

Fig. S5 shows the many-body spectra obtained using the Hamiltonian given by Eq. 3 in the main article with states from only sector B (8 states) and with all three sectors A, B, and C (14 states). Similar to the $N_{\mathrm{Tr}} = 2$ case, inclusion of the inter-triangulene states increases the splitting between singlet and triplet, and triplet and quintuplet. One can also see the order of total spin states (we show only the ten lowest energy states for the 8 state calculation) agrees with the order of states in BLBQ model for the lowest five states, compared with Fig. S6. The sixth state in the extended Hubbard model calculations with 14 states has $S = 2$ (seventh has $S = 0$). We attribute these differences between this extended Hubbard model and the BLBQ model to a CAS that is not large enough to converge excited states in this extended Hubbard model calculation.

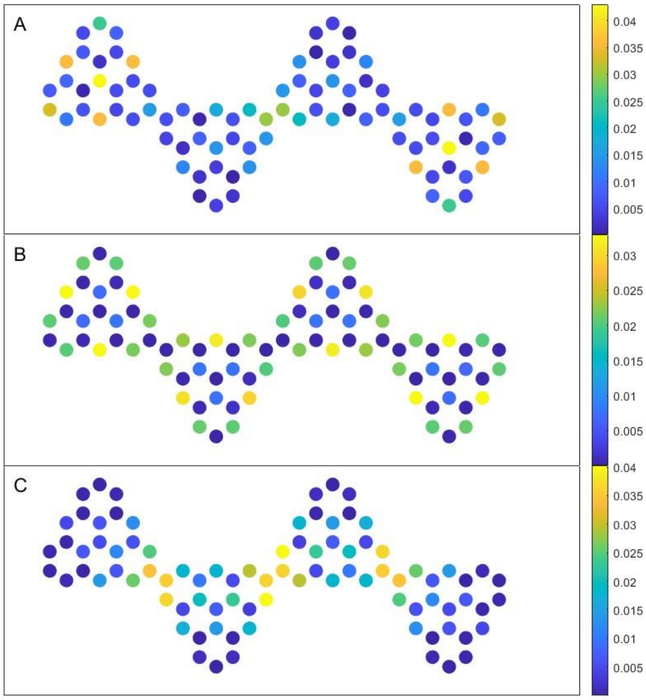

FIG. S4. Charge densities of Hartree-Fock spectrum from extended Hubbard model for $N_{\mathrm{Tr}} = 4$ in three energy sectors indicated in Fig. S3.

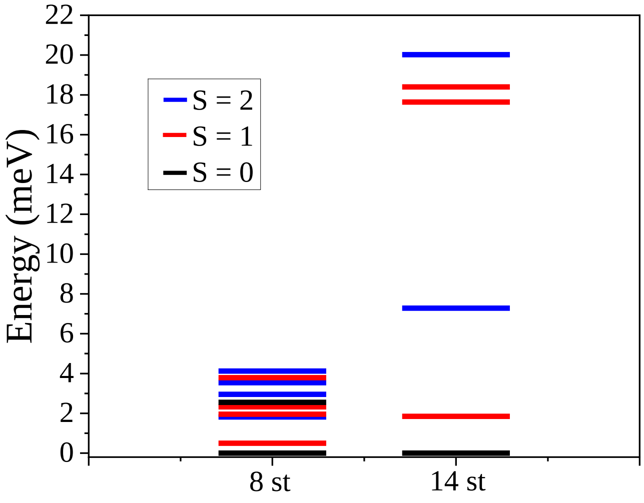

FIG. S5. Many-body spectrum from extended Hubbard model for $N_{\mathrm{Tr}} = 4$ for CAS(8,8) and CAS(14,14).

VII. SPIN MODEL RESULTS

The antiferromagnetic spin-1 Heisenberg model with the added biquadratic operator can effectively describe the chain of triangulane molecules. The effective Hamiltonian is:

$$H = J\sum_i [(\bar{S}_i \cdot \bar{S}_{i+1}) + \beta(\bar{S}_i \cdot \bar{S}_{i+1})^2], \tag{7}$$

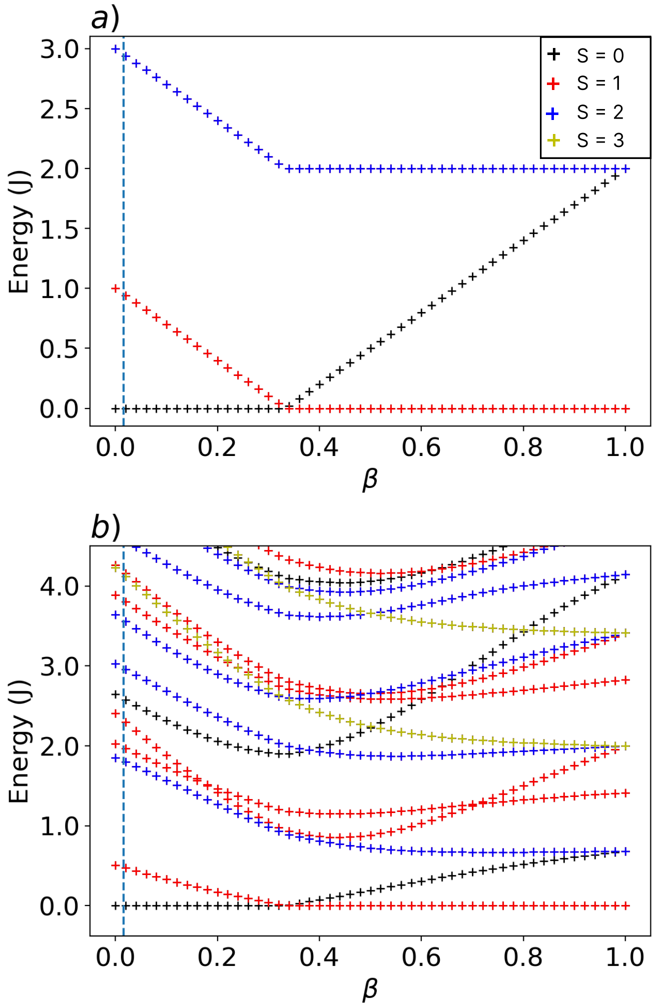

FIG. S6. The BLBQ model results as a function of β parameter for spin chains with (a) $N = 2$ and (b) $N = 4$ sites. The blue dashed line indicates the spectra from the Fermionic model in the main text.

where J is the coupling constant, β is the biquadratic term amplitude, and $\bar{S}_i$ is a spin vector of the ith molecule in the chain.

Using the definition of the ladder operators $\hat{S}_i^{\pm} = \hat{S}_i^x \pm i\hat{S}_i^y$, one can rewrite the Hamiltonian as:

$$\begin{aligned} H = J\sum_i &[\frac{1}{2}(\hat{S}_i^+\hat{S}_{i+1}^- + \hat{S}_i^-\hat{S}_{i+1}^+) + \hat{S}_i^z\hat{S}_{i+1}^z \\ &+ \frac{\beta}{4}(\hat{S}_i^+\hat{S}_{i+1}^-\hat{S}_i^+\hat{S}_{i+1}^- + \hat{S}_i^+\hat{S}_{i+1}^-\hat{S}_i^-\hat{S}_{i+1}^+ \\ &+ \hat{S}_i^-\hat{S}_{i+1}^+\hat{S}_i^-\hat{S}_{i+1}^+ + \hat{S}_i^-\hat{S}_{i+1}^+\hat{S}_i^+\hat{S}_{i+1}^-) \\ &+ \frac{\beta}{2}(\hat{S}_i^+\hat{S}_{i+1}^-\hat{S}_i^z\hat{S}_{i+1}^z + \hat{S}_i^-\hat{S}_{i+1}^+\hat{S}_i^z\hat{S}_{i+1}^z \\ &+ \hat{S}_i^z\hat{S}_{i+1}^z\hat{S}_i^+\hat{S}_{i+1}^- + \hat{S}_i^z\hat{S}_{i+1}^z\hat{S}_i^-\hat{S}_{i+1}^+) \\ &+ \beta(\hat{S}_i^z\hat{S}_{i+1}^z)^2]. \end{aligned} \tag{8}$$

The single spin-1 site has three possible $\hat{S}_i^z$ projections:

$|\uparrow\rangle = |1\rangle$, $|\downarrow\rangle = |-1\rangle$, and $|0\rangle$. To obtain analytical results, we construct the basis for N=2 chain sites, composed of all combinations of $\hat{S}_i^z$ values. The $\hat{S}^z = 0$ subspace is spanned by three basis vectors: $|0,0\rangle$, $|-1,1\rangle$, $|1,-1\rangle$. Acting with the Hamiltonian operator on this subspace results in:

$$\hat{H}\,|-1,1\rangle = J(1-\beta)\,|0,0\rangle + J\beta\,|1,-1\rangle + J(2\beta-1)\,|-1,1\rangle\,,$$
$$\hat{H}\,|0,0\rangle = 2J\beta\,|0,0\rangle + J(1-\beta)\,|1,-1\rangle + J(1-\beta)\,|-1,1\rangle\,,$$
$$\hat{H}\,|1,-1\rangle = J(1-\beta)\,|0,0\rangle + J(2\beta-1)\,|1,-1\rangle + J\beta\,|-1,1\rangle\,, \quad (9)$$

which gives us the matrix:

$$H_{S^z=0} = J\begin{bmatrix} (2\beta-1) & (1-\beta) & \beta \\ (1-\beta) & 2\beta & (1-\beta) \\ \beta & (1-\beta) & (2\beta-1) \end{bmatrix}. \quad (10)$$

Similarly, Hamiltonian matrices for other S^z subspaces are:

$$H_{|S^z|=1} = J\begin{bmatrix} \beta & 1 \\ 1 & \beta \end{bmatrix}$$
$$H_{|S^z|=2} = J\left[(1+\beta)\right]. \quad (11)$$

The full Hamiltonian matrix is block diagonal, and its eigenvalues for the singlet, triplet, and quintuplets are $E(S) = -2J(1-2\beta)$, $E(T) = -J(1-\beta)$, and $E(Q) = J(1+\beta)$. After shifting eigenvalues by $J(1+\beta)$, we see that only the singlet state depends on the β: $E(S) = -3J(1-\beta)$, $E(T) = -2J$, and $E(Q) = 0$. For $\beta < \frac{1}{3}$ the ground state is a singlet and for $\beta > \frac{1}{3}$ it is a triplet state. For $\beta = \frac{1}{3}$, the singlet and triplet states are degenerate, as shown in Fig. S6(a).

Fig. S6(b) shows numerical results for a chain of $N = 4$ spin-1 sites. The transition between the singlet and triplet ground state again occurs at $\beta = \frac{1}{3}$, but the gap between triplet and quintuplet now depends on β.

VIII. SUPEREXCHANGE MECHANISM ANALYSIS

Isolated triangulene has a triplet ground state and can be represented by an effective spin-1 site. We analyze the processes responsible for coupling between neighboring spin-1 states on the example of two triangulenes. Our methodology for treating Coulomb interaction relies on a two-step process. First, we include interactions at the Hartree-Fock level for a closed shell system, and next, we populate unoccupied states up to charge neutrality and diagonalize the many-body Hamiltonian within a restricted subspace. For the $N_{\mathrm{Tr}} = 2$ system, after the HF basis rotation, the degenerate shell states from two triangles form symmetric and antisymmetric linear combinations of states from each triangle, as seen in the wavefunctions of Fig. 3 of the main article. Moreover, we observe strong mixing between the four degenerate shell states and valence and conduction band inter-triangulene states.

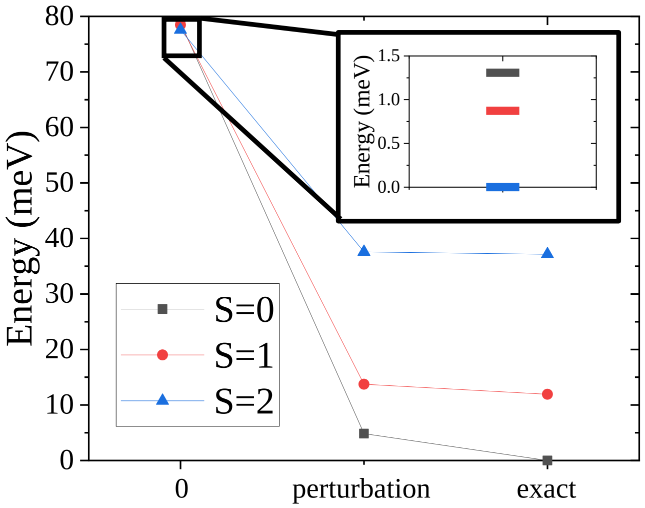

FIG. S7. Perturbation analysis of coupling between spins. 0 indicates the lowest energy total spin states obtained from diagonalization of many-body Hamiltonian matrix within a single occupation subspace of the degenerate shell states. Perturbation indicates energies of these states after energy corrections included within 2nd order perturbation theory. Exact are energies obtained after diagonalization full many-body Hamiltonian.

This hinders the perturbative analysis. The two isolated triangulenes have four perfectly degenerate edge states, two for each triangulene. The perfect strategy would be to rotate the HF degenerate shell states back to the isolated triangulene basis states. In that case, one could show that coupling between triangulene spin-1 states are through the inter-triangulene states - an indirect superexchange mechanism, one of the main results of this work. However, after HF self-consistent calculations, the degenerate shell states are too strongly hybridized with the inter-triangulene states.

We analyze the superexchange mechanism at the many-body level. We take six HF states to construct our many-body Hilbert space, the highest valence band state, four degenerate shell states and the lowest conduction band state. These states are labelled 5-10 in Fig. 2(c) of the main text, in this analysis, for simplicity we shift the labels to 1-6. The charge neutral system has $N_{\mathrm{el}} = 6$ electrons. For $S_{\mathrm{z}} = 0$, one can construct in total, $N_{\mathrm{cf}} = 400$ configurations. The low-energy subspace, which we call later a single occupation subspace, corresponds to double occupation of the valence band state and single occupation of four degenerate shell states. One can construct six such low-energy configurations. In the

occupation representation, these states can be written as

$$\begin{aligned} |I\rangle &= |1\downarrow, 1\uparrow, 2\downarrow, 3\downarrow, 4\uparrow, 5\uparrow\rangle \\ |II\rangle &= |1\downarrow, 1\uparrow, 2\uparrow, 3\uparrow, 4\downarrow, 5\downarrow\rangle \\ |III\rangle &= |1\downarrow, 1\uparrow, 2\downarrow, 3\uparrow, 4\downarrow, 5\uparrow\rangle \\ |IV\rangle &= |1\downarrow, 1\uparrow, 2\uparrow, 3\downarrow, 4\uparrow, 5\downarrow\rangle \\ |V\rangle &= |1\downarrow, 1\uparrow, 2\downarrow, 3\uparrow, 4\uparrow, 5\downarrow\rangle \\ |VI\rangle &= |1\downarrow, 1\uparrow, 2\uparrow, 3\downarrow, 4\downarrow, 5\uparrow\rangle, \end{aligned} \tag{12}$$

where state 1 is the valence band state lying just below the four degenerate shell states, states $2-5$ are the four degenerate shell states, and state 6 is the state just above the four degenerate states (unoccupied here). The six configurations defined in Eq. 12 can then be rotated into two total spin $S=0$ singlets, three $S=1$ triplets and one $S=2$ quintuplet. We obtain the lowest energy state $E_0(S)$ within each total spin single occupation subspace by diagonalizing a 2×2 matrix within $S=0$ subspace and a 3×3 matrix within $S=1$ subspace, at the same time appropriately rotating the full Hilbert space within each total spin sector. This procedure is related to the strong coupling between configurations given by Eq. 12, due to the hybridization between the degenerate shell states and inter-triangulene states. Next, the obtained lowest energy states within each total spin subspace, are corrected by second order perturbation contributions due to the coupling to the rest of states $E_i(S)$ from the many-body Hilbert space (which have been rotated to the total spin subspaces), beyond the single occupation subspace ($S=0$ subspace contains in total $N_{\mathrm{cf}}=175$ configurations, $S=1$ contains $N_{\mathrm{cf}}=189$ configurations, and $S=2$ contains $N_{\mathrm{cf}}=35$ configurations). The perturbative Hamiltonian is written as

$$E^{(2)}(S)=\sum_{i=1}^{N_{\mathrm{cf}}(S)}\frac{|\langle\Psi_0(S)|H|\Psi_i(S)\rangle|^2}{E_0(S)-E_{\mathrm{i}}(S)}, \tag{13}$$

where Ψ_0 and Ψ_i are the many-body wave function corresponding to energy E_0 and E_i, respectively, and H is the many-body Hamiltonian given by Eq. (3) in the main article. Although this procedure is second order in perturbation theory, if the hybridization between the edge states of the individual triangulenes and the inter-triangulene states were weak, then perturbative treatment of the lowest energy total spin states (corresponding to singlet, triplet and quintuplet) would be appropriate. In that case, fourth order perturbation theory would be required in order to describe coupling of the degenerate states of two triangulenes (and thus the spin-1 quasiparticles) through the inter-triangulene states.

The energy spectra after diagonalization within the single occupation subspace, and after second order perturbation correction is compared to the exact many-body spectrum in Fig. S7. The order of total spin states after basis rotation within a single occupation subspace is the same as within truncated $N_{\mathrm{st}}=4$ states with the $S=2$ quintuplet as the ground state, see Fig. 4(a) in the main article. Coupling of the single occupation subspace to higher energy configurations leads to a change of the order of states that now agrees with predictions within the two spin-1 Heisenberg Hamiltonian, with the singlet as the ground state. Furthermore, results after this coupling, agrees as well as with the experiments[21]. The energies are close to the exact energies obtained after diagonalization of the full many-body Hamiltonian. We notice that configurations mainly contributing to the perturbation includes all six HF states, thus both the state below and the state above the four degenerate shell states are important, which confirms the existence of an indirect AFM superexchange mechanism.

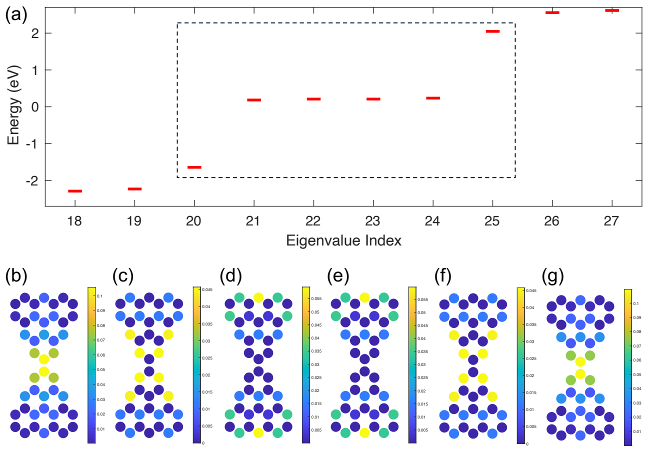

FIG. S8. (a) Tight-binding spectrum for the triangulene dimer. (c-f) show the corresponding charge densities for the states in the box labelled 20-25 in (a).

IX. THE SINGLE SLATER DETERMINANT RESTRICTION (HOPPING INTEGRALS IN A TRIANGULENE DIMER)

The triangulene dimer (TD) shown in Fig. 1 of the main text behaves as two spin-1 sites with antiferromagnetic coupling - thus the ground state is a singlet. The failure of mean-field methods to properly capture the ground state lies in the fact that the singlet ground state cannot be written as a single Slater determinant. This raises the question of how does one reliably obtain effective hopping parameters for a triangulene spin chain when more than one Slater determinant is required to describe the ground state. In the ideal situation one would develop a tight-binding model that fits very well with DFT calculations at half-filling, a subtle and tricky problem due to the nature of the ground state in this system.

In order to obtain position dependent hopping integrals shown in Fig. 3 of the main article, we perform Hartree-Fock calculations for the charged system with an empty shell at the Fermi level (the system has +4e charge), and in the last step of self-consistent calculation we add back four electrons to the two lowest states from

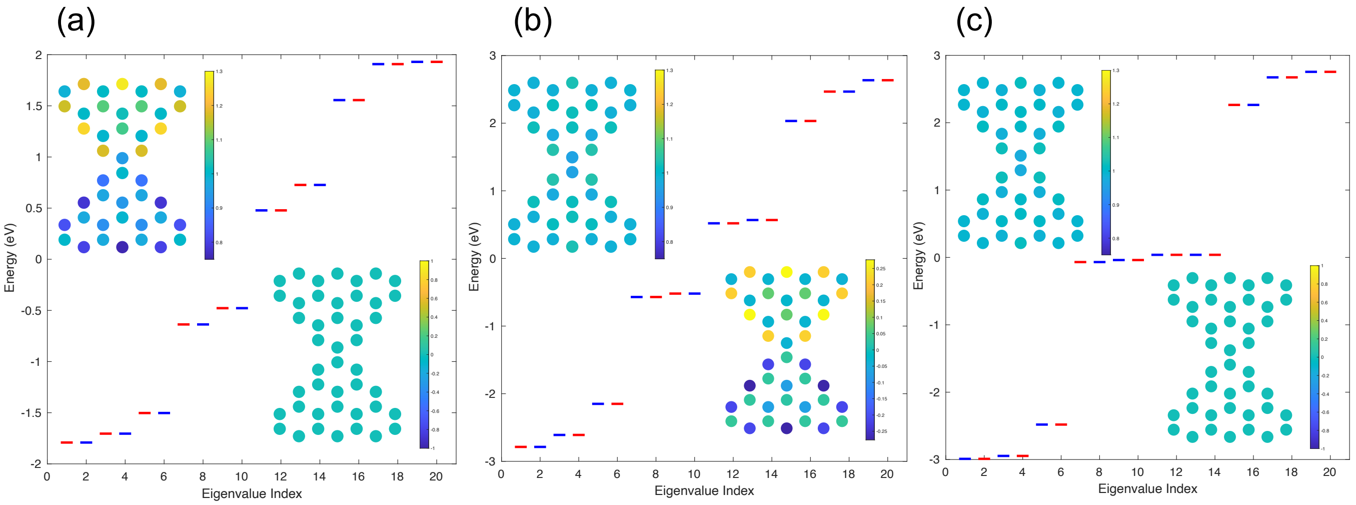

FIG. S9. HF spectrum for the (a) charge broken symmetry state (b) spin broken symmetry state and (c) fractionally filled shell state. Red levels correspond to spin up and blue levels correspond to spin down. The upper left inset corresponds to the ground state charge density with the color bar representing the average number of electrons per site. The bottom left corresponds the spin density with the color bar representing the average spin per site.

the degenerate shell to make the system charge neutral. Next, we rotate these Hartree-Fock states at charge neutrality to a tight-binding basis, obtaining a single-particle Hamiltonian with position dependent hoppings.

In this section, we discuss, in more detail, the issue of how to obtain an alternate HF basis at half-filling in order to get position dependent hoppings that confirm the validity of our approach. To better understand the procedure, we briefly analyze the TB spectrum for the TD shown in Fig. 1. We begin with the TB Hamiltonian given by

$$H_{TB} = \sum_{i,l,\sigma} t_{il\sigma} c^{\dagger}_{i\sigma} c_{l\sigma}, \tag{14}$$

where the hopping parameters $t_{il\sigma}$ are defined in the main text. We note this Hamiltonian represents the geometry of the triangulene dimer but assumes the hopping elements to be only distance dependent and equal to the infinite graphene values. The corresponding TB wavefunctions can be written as

$$\psi^{TB}_{\alpha}(\vec{r}) = \sum_{i,\sigma} a^{\alpha}_{i\sigma} \phi_i(\vec{r}), \tag{15}$$

where $\phi_i(\vec{r})$ are defined in Eq. 2 and $a^{\alpha}_{i\sigma}$ are expansion coefficients obtained by solving the TB Hamiltonian.

The TB solution simply serves as an initial guess to the HF calculations that will follow. Fig. S8(a) shows the TB spectrum. We see there are four states near the Fermi level (at half-filling) with two of the states (labelled 22 and 23) being degenerate. Fig. S8(c-e) shows the corresponding charge densities for these states. We note that at half-filling there is a question of how to occupy the two degenerate states (22 and 23) at the Fermi level. We will now address this issue.

We now analyze the solution of Eq. 1 of the main text for a half-filled (charge neutral) system with $S_z = 0$. We note a subtlety that in this calculation $\kappa = 6$ (in contrast to $\kappa = 3$ used in the main article), for simpler convergence - this will not be important as this calculation is just for demonstrative purposes. We first consider occupying all states up to the Fermi level and thus doubly occupy states labelled 21 and 22 in Fig. S8(a). Fig. S9(a) shows the HF spectrum along with the corresponding charge density and spin density of the HF ground state. We observe that this state has accumulated charge on one triangulene molecule and has uniform spin density. Another state exists with the exact same energy in which the charge accumulation is on the lower triangulene rather than the upper triangulene. Furthermore, we notice the spin density is completely uniform, contrary to the expectation that the spins should order anti-ferromagnetically on the two triangulenes. We note that the effective hopping computed using Eq. 2 of the main text are again normalized and the effective hopping t_3 connecting the two triangulenes is reduced. In fact, due to the charge broken symmetry, the hoppings are also different on one triangulene than the other. We note an issue for using this HF state, is that, another (single Slater determinant) state exists which has a net charge density on the other triangulene which is exactly degenerate with this state. This state is not captured here, and thus CI calculations from this starting point fail as trying to construct excitations from this state would fail to capture excitations from it's degenerate counter part.

An alternate method to solve the HF equation is to properly form linear combinations of the degenerate TB states defined in Eq. 15 near the Fermi level (states 21 and 22) in Fig. S8(a). Thus we create two new states

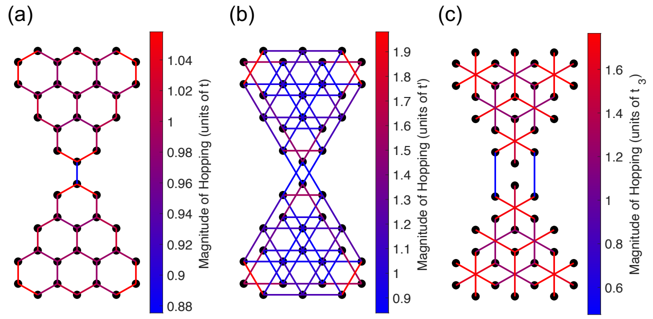

FIG. S10. Effective HF hopping elements for (a) nearest neighbor t (b) next nearest neighbor t' (c) next-next nearest neighbor t_3 for the HF calculation with the fractionally filled shell. The black dots correspond to the location of carbon atoms, and the line connecting them corresponds to the effective hopping between carbon atoms. The units for each color plot are measured with respect to their 2D infinite system hopping values.

with the following rotation:

$$\psi_{\pm}^{TB}(\vec{r}) = \frac{1}{\sqrt{2}} \left(\psi_{21}^{TB}(\vec{r}) \pm \psi_{22}^{TB}(\vec{r}) \right), \qquad (16)$$

This will form states localized purely on one triangulene or another. Then, in the HF procedure, in our initial guess we singly occupy these states putting one electron on the state "+" and another on the state "−" defined in Eq. 16. This will break the spin uniformity of the triangulene dimer, as one triangulene will be occupied with one extra spin up electron and the other with one extra spin down electron, thus generating a spin broken symmetry Slater determinant. We note this calculation is done for $\kappa = 6$ as well. Fig. S9(b) shows the solution of the HF equation in this broken symmetry situation. We see that we converge to a state that has charge uniformity, and shows AFM ordering of spins. In this HF state, the effective hoppings are again non uniform and in fact, spin dependent due to the spin broken symmetry nature of the state. We also observed, again, the effective t_3 hopping connecting the two triangulenes is reduced.

Now, our goal is to obtain a single Slater determinant that preserves the symmetry of the TD. Occupation of the shell at the Fermi level is critical and one way to attack this problem is by considering a fractional occupation of the states near the Fermi level. By occupying (at each iteration of the HF procedure) each level with 1/2 a spin up particle and 1/2 a spin down particle and thus each level is equally treated. This yields the state shown in Fig. S9(c). We note, for this calculation $\kappa = 3$ consistent with the dielectric screening constant used in the paper. We emphasize that each of the degenerate shell states near the Fermi level is equally and fractionally occupied. We have spin degeneracy due to the the $S = 0$ character of this state. The spin-density is exactly uniform, and the charge density is close to uniform. This state is also a good starting point for CI calculations giving the same results as starting from a charged system instead (confirming our many-body calculations are independent of the chosen basis). Now the effective hopping elements are shown in Fig. S10. We observe, the effective hopping parameters are still inhomogeneous across the structure and the t_3 hopping connecting the two triangulene is still dramatically reduced.

X. REFERENCES

1 Ahlrichs, R., Bär, M., Häser, M., Horn, H. & Kölmel, C. Electronic structure calculations on workstation computers: The program system turbomole. *Chem. Phys. Lett.* **162**, 165–169 (1989).

2 Dirac, P. A. M. Quantum mechanics of many-electron systems. *Proc. Royal Soc. (London) A* **123**, 714–733 (1929).

3 Slater, J. C. A simplification of the hartree-fock method. *Phys. Rev.* **81**, 385 (1951).

4 Vosko, S. H., Wilk, L. & Nusair, M. Accurate spin-dependent electron liquid correlation energies for local spin density calculations: a critical analysis. *Can. J. Phys.* **58**, 1200–1211 (1980).

5 Becke, A. D. Density-functional exchange-energy approximation with correct asymptotic behavior. *Phys. Rev. A* **38**, 3098 (1988).

6 Lee, C., Yang, W. & Parr, R. G. Development of the colle-salvetti correlation-energy formula into a functional of the electron density. *Phys. Rev. B* **37**, 785 (1988).

7 Becke, A. D. Density-functional thermochemistry. i. the effect of the exchange-only gradient correction. *J. Chem. Phys.* **96**, 2155–2160 (1992).

8 Weigend, F. Accurate coulomb-fitting basis sets for h to rn. *Phys. Chem. Chem. Phys.* **8**, 1057–1065 (2006).

9 Grimme, S., Antony, J., Ehrlich, S. & Krieg, H. A consistent and accurate ab initio parametrization of density functional dispersion correction (dft-d) for the 94 elements h-pu. *J. Chem. Phys.* **132** (2010).

10 Eichkorn, K., Treutler, O., Öhm, H., Häser, M. & Ahlrichs, R. Auxiliary basis sets to approximate coulomb potentials. *Chem. Phys. Lett.* **240**, 283–290 (1995).

11 Eichkorn, K., Weigend, F., Treutler, O. & Ahlrichs, R. Auxiliary basis sets for main row atoms and transition metals and their use to approximate coulomb potentials. *Theor. Chem. Acc.* **97**, 119–124 (1997).

12 Sierka, M., Hogekamp, A. & Ahlrichs, R. Fast evaluation of the coulomb potential for electron densities using multipole accelerated resolution of identity approximation. *J. Chem. Phys.* **118**, 9136–9148 (2003).

13 Noodleman, L. Valence bond description of antiferromagnetic coupling in transition metal dimers. *J. Chem. Phys.* **74**, 5737–5743 (1981).

14 Güçlü, A. D., Potasz, P., Korkusinski, M., Hawrylak, P.

et al. Graphene quantum dots (Springer, 2014).

[15] Ransil, B. J. Studies in molecular structure. ii. lcao-mo-scf wave functions for selected first-row diatomic molecules. *Rev. Mod. Phys.* **32**, 245–254 (1960). URL `https://link.aps.org/doi/10.1103/RevModPhys.32.245`. (Accessed 2023-12-04).

[16] Galassi, M. *et al. GNU scientific library* (Network Theory Limited Godalming, 2002).

[17] Lieb, E. H. Two theorems on the hubbard model. *Phys. Rev. Lett.* **62**, 1201–1204 (1989). URL `https://link.aps.org/doi/10.1103/PhysRevLett.62.1201`. (Accessed 2023-12-04).

[18] Pavliček, N. *et al.* Synthesis and characterization of triangulene. *Nature Nanotechnology* **12**, 308–311 (2017). URL `https://doi.org/10.1038/nnano.2016.305`. (Accessed 2023-12-04).

[19] Mishra, S. *et al.* Synthesis and characterization of π-extended triangulene. *Journal of the American Chemical Society* **141**, 10621–10625 (2019). URL `https://doi.org/10.1021/jacs.9b05319`. (Accessed 2023-12-04).

[20] Ortiz, R., Catarina, G. & Fernández-Rossier, J. Theory of triangulene two-dimensional crystals. *2D Materials* **10**, 015015 (2022).

[21] Mishra, S. *et al.* Observation of fractional edge excitations in nanographene spin chains. *Nature* **598**, 287–292 (2021). URL `https://doi.org/10.1038/s41586-021-03842-3`. (Accessed 2023-10-13).
